# Adolescent Sense of Coherence over a Four-Year Period and the Pandemic: Junior and Senior High School Students Enrolled Before and After the Pandemic Broke out in Japan

**DOI:** 10.3390/bs15040504

**Published:** 2025-04-09

**Authors:** Tomoko Omiya, Naoko Kumada Deguchi

**Affiliations:** 1 Public Health Nursing, Institute of Medicine, University of Tsukuba, Tsukuba 305-8575, Japan; 2 Faculty of Education, Shizuoka University, Shizuoka 422-8017, Japan; naokok-tky@umin.ac.jp

**Keywords:** adolescent students, COVID-19 pandemic, sense of coherence, sense of school belonging

## Abstract

Based on Antonovsky’s theory, we explored the importance of adolescent sense of coherence (SOC) in coping with stress and how it was affected by the pandemic. Using longitudinal data from junior and senior high school students in urban areas in Japan, we examined the trends in SOC and factors related to SOC in students enrolled before and after the COVID-19 pandemic. With the cooperation of the Tokyo Metropolitan Secondary Education School, we surveyed 96 students who enrolled in 2018 (G1) and 144 students who enrolled in 2020 (G2). Four surveys were conducted for G1 and three for G2. Survey items included SOC, psychosomatic symptoms scale, Athens insomnia scale, school belonging scale, and stress experience scale. We followed the trends in SOC scores by gender and performed *t*-tests and multiple regression analysis. G2 had higher baseline SOC scores than G1, but the significant difference between the two groups disappeared by 2022. From 2019, comprehensibility and manageability significantly increased in G1 for girls, but meaningfulness decreased in G2 for both boys and girls. Multiple regression analysis showed no correlation between baseline SOC and SOC in 2022 in G1, which differed from G2, suggesting that the pandemic may have changed their perception of the world.

## 1. Introduction

The COVID-19 pandemic has been an unprecedented experience for anyone worldwide. Initially, there were no prophylactic or specific drugs against COVID-19, and the unknown virus mutated easily, making people fearful and indecisive. In Japan, the COVID-19 pandemic has caused major disruptions to the lives of junior and senior high school students, including the unprecedented nationwide simultaneous closure of schools in March 2020 (Ministry of Education, Culture, Sports, Science and Technology, 2020). In Japan, the 11th wave (the 11th peak in the number of infected people) was reported to have struck in the summer of 2024 (Japan Science and Technology Agency, 2024), and as of January 2025, there are still constant reports of infections.

Situations such as the COVID-19 pandemic, in which people are uncertain of their infection status, feel helpless, and struggle to determine what the future might hold, cause anxiety. The prolonged closure of social life and spread of life-threatening infectious diseases are extremely stressful and are thought to reduce one’s sense of coherence (SOC), a powerful stress-coping mechanism. SOC is the core concept of health generation theory proposed by Antonovsky and consists of three subscales: comprehensibility, manageability, and meaningfulness (Antonovsky, 1987, 1996). SOC is the ability to cope with stress and generate health, not only to protect oneself from the stressors and crises that are present in people’s lives, but also to drive growth and development, and nurture a rich life with joy, sorrow, and happiness. Thus, it is considered the ultimate health factor in life (Yamazaki & Togari, 2011). It has been shown that in middle-aged men, a lower SOC during long-term follow-up is significantly associated with a higher risk of all-cause mortality (Piiroinen et al., 2020), and that a weak SOC increases the risk of death in the general adult population (Yamauchi et al., 2020). In addition, systematic reviews have revealed that SOC is a powerful predictor of health-related indicators, with strong SOC consistently shown to function as a protective factor against depression, burnout, and hopelessness (Piiroinen et al., 2022).

According to Antonovsky (1987, 1996), adolescent SOC is highly vulnerable and varies widely. Japanese adolescents were reported to be unwell during the pandemic period (Okajima et al., 2022; Okuyama et al., 2021; Sasaki et al., 2022). Hosozawa et al. (2024) used longitudinal survey data from 2000 16-year-old children in the Tokyo area to examine the impact of the pandemic on their mental health. They reported that there were differences in mental health changes by gender, with girls’ mental health worsening during the early school closure period and boys’ depressive symptoms worsening in 2020–2021, the second year of the pandemic (Hosozawa et al., 2024). Studies on adolescent SOC during the pandemic are limited. Omiya et al. (2021) measured the SOC of middle school students at two points, before and during the pandemic, and found that SOC increased after the pandemic broke out, although 40% of those who declined did not have a sense of “belonging at school”. Tal-Saban and Zaguri-Vittenberg (2022) found that SOC among 13–18-year-olds was high during the outbreak, but their quality of school life was low. Although adolescent SOC is an important indicator related to suicide and mental health deterioration and is said to be an important predictor of SOC in adulthood (Feldt et al., 2005; Volanen et al., 2006), to our knowledge, no other studies have examined SOC in adolescents during pandemics.

In Japan, schools were closed for only a few months in the early stages of the pandemic, but measures such as wearing masks, banning conversations during lunch, and ensuring social distancing continued for a long time afterward. In particular, wearing masks in school makes it difficult to recognize facial expressions, which negatively affects the quality of communication and relationships (Spitzer, 2020). When considering adolescents, stressful experiences related to school demands, relationships with friends, and club activities should also be considered.

DeVylder et al. (2024) reported that mental disorders in adolescents, which had decreased before the pandemic, began to rise again at the same time as the pandemic, despite the low infection rate of COVID-19 in Tokyo, Japan. They attributed this to COVID-19-related social and environmental factors. In addition, Raney et al. (2022) revealed that children with more adverse experiences related to the pandemic had longer screen time and less physical activity. Orben et al. (2020) stated that although the measures to maintain physical distance from others due to the pandemic are temporary, the impact of maintaining physical distance for several months may be stronger on young people’s development than on adults, as this account constitutes a large proportion of their lives during a sensitive period of development. They were concerned about the impact of the pandemic on young people’s development. In addition, many studies on adolescents were conducted during the first months of the pandemic (i.e., through the summer of 2020) (Barendse et al., 2023; Hafstad et al., 2021). However, the impact of the pandemic on adolescents may evolve over time, influenced by factors such as the reopening of schools or changes in social distance regulations (Hosozawa et al., 2024), and should be tracked over time. Furthermore, the significance of the pandemic and school closure is likely to be different for the group that experienced school closure while still in school and the group entering school in April 2020, where the pandemic coincided with their enrollment. Those still in school were unable to attend important ceremonies and had difficulty building relationships with friends through online classes and other means. If the impact of the pandemic differs between the group that experienced the pandemic after enrollment and the group that enrolled after the pandemic, interventions and support will only be effective if they reflect the circumstances of each group. However, no studies have been conducted from such a perspective. It is important to clarify the characteristics of these two groups to obtain suggestions for support for each.

How has SOC been affected by the pandemic among unstable adolescents? The purpose of this study was to examine the trends in SOC and the factors associated with SOC for students enrolled before and after the pandemic, using longitudinal data for junior high and high school students.

## 2. Materials and Methods

### 2.1. Participants

Cooperation was obtained from the Tokyo Metropolitan Secondary School A (a six-year integrated junior and senior high school). One of the authors (NKD) worked at School A as a Yogo-teacher (a role similar to that of a school nurse) until March 2018. However, by the time the survey began, NKD had retired and was no longer acquainted with the students. Meetings with the school and the survey were conducted by OT. Based on the above, there was no potential conflict of interest. The participants were 96 students who enrolled in 2018 (Group 1: G1) and 144 students who enrolled in 2020 (Group 2: G2), with G1 responding to all surveys from 2019 to 2022, and G2 responding to all surveys from 2020 to 2022. In February 2020 in Japan, a state of emergency was declared due to COVID-19, and all schools were closed for approximately three months, which means that G2 entered school during COVID-19. Students’ gender and age were recorded as basic demographic attributes.

The baseline for G1 was spring 2019, while for G2 it was summer 2020. G1 data were collected through four surveys over four years, from eighth through eleventh grade, whereas G2 data were gathered through three surveys over three years, from seventh to ninth grade.

### 2.2. Ethical Considerations

This study was conducted according to the guidelines of the Declaration of Helsinki. First, because the participants were minors, their parents were given a written outline of the survey, their written consent was obtained, and the survey instrument was distributed only to students whose parents provided consent. We explained the program to the students in writing and confirmed their willingness to participate through a questionnaire. We also explained that participation in the survey was voluntary, there was no disadvantage to not participating, and that participants could withdraw at any time. As this was a longitudinal study, each student was assigned a random six-digit personal ID number to manage their participant data. Documents linking personal ID numbers to student names were maintained by one person in charge of each school and archived so that the individuals could not be identified from their responses. Student surveys were distributed by teachers at schools and collected in boxes. They were distributed to parents through the students and collected. This study was approved by the Medical Ethics Review Committee of the University of Tsukuba (approval numbers: 1343, 1343-1 and 2); approval dates: 30 January 2019, 10 June 2020, and 8 June 2021).

### 2.3. Sense of Coherence

We used the sense of coherence 13-item scale (SOC-13), a shortened Japanese version of the original SOC 29-item scale developed by Antonovsky. The reliability and validity of the SOC-13 were confirmed by Togari and Yamazaki through a survey conducted in Japan (Togari et al., 2007). The SOC-13 comprises three subscales: comprehension (five items), manageability (four items), and meaningfulness (four items). In the SOC-13 scale, items are rated on a 5-point Likert scale. Higher scores indicate a greater ability to maintain health and cope with stress. G1 was measured four times from 2019 to 2022, and G2 was measured three times from 2020 to 2022. The Cronbach’s SOC-13 alphas in this study ranged from 0.84 to 0.87.

### 2.4. School Membership Scale

We used the school membership scale, a shortened Japanese version of the psychological sense of school membership scale (Goodenow, 1993) developed by Goodenow, an educational psychologist (Togari et al., 2011). The scale consists of three components—student acceptance (five items), teacher acceptance (four items), and sense of belonging (four items)—with a total of 13 items measured using a five-item method. The reliability and validity of the Japanese version of the scale have been confirmed (Soldatos et al., 2000). Higher total scores indicated a greater commitment to school life. G1 was measured four times from 2019 to 2022, and G2 was measured three times from 2020 to 2022. The Cronbach’s alpha in this study ranged from 0.81 to 0.89.

### 2.5. Baseline Measures (2019 for G1 and 2020 for G2)

#### Subjective Physical Health

They were asked about six psychosomatic symptoms as perceived by Ben-Sira (1982) (headache, abdominal pain, difficulty sleeping, palpitations, vertigo or dizziness, irritability) and two items relating to diarrhea/constipation and body pain (e.g., shoulder pain). Respondents were asked to answer “always”, “sometimes”, “not often”, and “not at all”, using a 4-point scale, with 1 to 4 points given for each. Scores range from 8 to 32 points, with higher scores indicating better physical condition.

A universal scale for determining insomnia, created by the World Project on Sleep and Health, was established by the World Health Organization (Soldatos et al., 2000) and assesses the quality and duration of sleep. It consists of eight questions with a maximum score of 24. Higher scores indicate greater insomnia. The reliability and validity of the Japanese version of the scale have been verified (Okajima et al., 2013). The Cronbach’s alpha coefficient was 0.79.

### 2.6. Scale Measured in 2022

#### Students’ Perceptions of Stressful Experiences

The reliability and validity of the High School Student Life-Related Stress Scale, developed by Ishida et al. (2017), were confirmed in assessing adolescents’ perceptions of student life-related stress. The developer approved the adaptation of the scale for use with middle school students. The scale consists of five domains: (1) teachers, (2) club activities, (3) academic performance, (4) friends and opposites, and (5) family. Each domain contains four to five items, for a total of 19 items in all domains. Participants responded on a 4-point rating scale (1 = never experienced stress, 2 = occasionally experienced stress, 3 = experienced stress, and 4 = frequently experienced stress). We asked about negative perceptions of school life, such as “My classmates ignored me and made me feel uncomfortable”. Based on discussions with the authors of this study and previous research (Okayasu et al., 1992), we also included five stressors related to club activities, because they are important for Japanese junior high school students. Therefore, we used an adapted version of this scale, which consists of 24 items rated on a 4-point Likert scale. Data measured in 2022 were used for G1 and G2. The Cronbach’s alpha ranged between 0.80 and 0.91.

Method of analysis: The number of people was categorized by gender for the baseline data (2019 and 2020) for G1 and G2. Mean scores and standard deviations were calculated for three constructs: subjective psychosomatic symptoms measured at baseline, Athens insomnia score, SOC (total and per subscale), and school membership scale. For 2022, the mean scores and standard deviations were calculated for SOC (total and per subscale), the three components of the school membership scale, and stress experiences. A *t*-test was performed between the two groups (G1 and G2).

Annual change scores were calculated for each SOC total score and subscale, stratified by G1 and G2, as well as by gender. G1 followed the score trends over a four-year period, from 2019 to 2022, while G2 followed them over a three-year period from 2020 to 2022. A one-way analysis of variance with repeated measures (corresponding factors) was performed on the annual scores by gender for G1 and G2. Multiple comparisons using Bonferroni corrections were conducted to test the main effects. The mean scores and standard deviations were also calculated by gender, and *t*-tests were performed. Similarly, for the school membership scale, repeated measures and Bonferroni’s tests were conducted for the total score and three subscales (student acceptance, teacher acceptance, and sense of belonging) by year for each group, using *t*-tests for gender comparisons.

Multiple regression analysis was performed for each group with the 2022 SOC total score as the dependent variable. To address missing data without reducing the sample size, multiple imputation (MI) was conducted. The explanatory variables were the baseline SOC total score, psychosomatic illness, Athens insomnia scale score, and the 2022 sense of school belonging scale and stress experience scale, after confirming that the Spearman’s ρ correlation with the dependent variable was significant in one or both groups. SPSS version 29 was used for statistical analysis.

## 3. Results

### 3.1. Group Characteristics and Data by Year for Each Group

As shown in Table 1, for the students in G1 (enrolled in 2018), 49 (51.0%) were boys and 47 (49.0%) were girls. The students in G2 (enrolled in 2020) included 73 boys (50.7%) and 71 girls (49.3%). The *t*-test results show significant differences between G1 and G2 in baseline subjective physical health (*p* = 0.001), total SOC and three subscales (*p* < 0.001), and the school membership scale subscale (*p* = 0.038). For all the above, G2 scored higher than G1. For the 2022 measurement score, no measurement variables showed significant differences between G1 and G2.

As shown in Table 2, A one-way ANOVA (with Bonferroni’s for subsequent tests) with repeated measures (factors with correspondence) by year, group, and gender was performed for the SOC total score and the three subscales. The results showed that, in G1, the SOC total score for girls was different in 2019 and 2020 (39.2 ± 6.8 and 43.8 ± 8.1, *p* < 0.001), and in 2019 and 2021 (42.7 ± 8.0, *p* = 0.023), showing significant differences. For the subscales, significant differences were found for comprehensibility in 2019 and 2020 (14.0 ± 3.0 and 16.1 ± 3.5, *p* = 0.001) and 2019 and 2021 (15.8 ± 3.4, *p* = 0.013) for girls. For manageability, girls showed a significant difference in 2019 (11.9 ± 2.9) and 2020 (13.2 ± 2.9) (*p* = 0.014). Boys showed a significant difference in 2019 (11.7 ± 2.6) and 2021 (13.0 ± 2.7) (*p* = 0.027).

A comparison of G1 gender scores showed a significant difference in the 2020 meaningfulness scores between boys and girls, with girls scoring higher (*p* = 0.001).

Looking at year-to-year trends by gender for G2, girls had the highest baseline scores in 2020 for the SOC total score, as well as the comprehensibility and meaningfulness subscales, with significant differences observed between 2020 and 2021, and between 2020 and 2022 (*p* < 0.001–0.043). Significant differences were found between 2020 and 2021, and between 2020 and 2022 (*p* < 0.001 to 0.043). For boys, significant differences were found only in the meaningfulness subscale, with higher scores in 2020 (15.0 ± 2.6) compared to 2021 (13.3 ± 3.3, *p* < 0.001) and 2022 (13.5 ± 2.8, *p* = 0.001). Regarding the comparison of G2 scores, no gender differences were found in the meaningfulness subscale in 2020 and 2021. However, all other measured variables were significantly higher in boys (*p* < 0.001 to 0.019).

As shown in Table 3, A one-way ANOVA (with Bonferroni’s test for subsequent tests) with repeated measures (corresponding factors) by year, group, and gender for the school membership scale showed no significant differences in annual scores for G1.

Comparisons by gender showed significantly higher scores for Accepted by students in 2019 (*p* = 0.004) and 2020 (*p* = 0.013), Accepted by Teachers in 2020 (*p* = 0.016) and 2022 (*p* = 0.023), and Belonging to school for girls in 2022 (*p* = 0.047). G1 showed no significant gender differences in annual scores. G2 showed that in the Belonging to school score, the 2020 score was significantly higher than the 2021 and 2022 scores for both boys and girls (*p* < 0.001 to 0.010). No year-to-year differences were found in the other measured variables. No significant gender differences were found for any of the three subscales in any year.

### 3.2. Factors Related to SOC After the Pandemic by Group

Table 4 presents the results of the multiple regression analysis using the 2022 SOC total score as the dependent variable. In G1, the sense of belonging (β = 0.516, *p* = 0.009), a subscale of the sense of school belonging scale, was positively and significantly associated with the dependent variable, while academic stress was negatively and significantly associated (β = −0.393, *p* = 0.010). In G2, the baseline total SOC score showed a significant positive association with the dependent variable (β = 0.370, *p* = 0.001), while it negatively and significantly correlated with the covariates gender, with girls having lower SOC (β = −0.212, *p* = 0.009) and family stress (β = −0.230, *p* = 0.015).

## 4. Discussion

### 4.1. G1 and G2 from the Perspective of Annual Trends in SOC

There was a significant difference in baseline SOC scores between G1 (enrolled before the pandemic) and G2 (enrolled during the pandemic). Since G2 scored significantly higher on the three subscales of the SOC scale, and the belonging to school subscale of the school membership scale, it is safe to conclude that they were admitted in good condition. However, because no significant differences were detected in the measured variables between the two groups in 2022, we do not believe it is appropriate to conclude that G1 and G2 are completely heterogeneous populations. In G2, it is reasonable to speculate that the impact of the pandemic and simultaneous school closures may have influenced baseline scores. In Japan, April is the beginning of the school year, and the entrance ceremony is held for the new school year. In G2, however, all schools had been closed nationwide because of the pandemic before the school year started (March 2020), and students did not attend school until June 2020. The survey was conducted between July and August 2020 with G2 participants, specifically immediately after they entered middle school. Expectations are high during the transition from elementary to middle school (Hiraga et al., 1996). In addition, the cooperating schools in this study held entrance exams, and students were enrolled in middle school. The fact that students have been reported to acquire a sense of self-growth and academic fulfillment through exams (Murai et al., 2022) can be used to explain the high SOC scores observed in G2 after the exam. A possible reason for the increase in G1 baseline and 2020 SOC scores could be the sense of “getting by” as school gradually reopened after the closure. This interpretation is supported by the significant increase in manageability from 2019 to 2020 for G1 girls. The results of this study are as follows: comprehensibility, the “confidence that I can understand my situation and make predictions about the future,” was significantly higher in 2020 and 2021 than in 2019 for G1 girls. Comprehensibility is fostered by “the experience of consistency”, as Antonovsky says (Yamazaki et al., 2008). School is a place where rules and norms are clear, and it is conceivable that students’ sense of comprehensibility was nurtured by the stability provided by these structures. For G1, there were no gender differences in scores except for the 2020 total score and meaningfulness, which remained stable overall, with slight or increasing trends over the four years after the school was closed. In contrast, the SOC in G2 showed a downward trend from high baseline values, especially among girls. However, as there was no significant difference between the scores for G1 and G2 in 2022, it can be interpreted that the scores for G2 were too high at the time of admission. In other words, for G2, it is conceivable that the exuberance of the immediate post-entry period subsided in daily life. However, it must be noted that meaningfulness for both boys and girls in G2 declined significantly from 2020 to 2022. Meaningfulness, one of the three subscales, is an important component of “confidence”. It expresses the idea that “I feel a sense of fulfillment and meaning in my daily activities. Meaningfulness also involves the ability to “find a new meaning” and respond flexibly to the constant changes in the world, nurtured through life experiences, such as “participating in shaping outcomes”. It is possible that G2, in the course of their restricted school life, felt that school life was different from what they had envisioned when they first entered school, and may have had difficulty finding meaning in the various changes.

### 4.2. Tracking the Changes in the School Membership Scale

On the school membership scale, girls scored significantly higher in G1, which is consistent with previous studies (Yamazaki et al., 2008). The scores were stable with no significant changes from year to year. In contrast, while there were no gender differences in G2, a significant decline in belonging to school was observed from the 2020 baseline for both boys and girls in 2021 and 2022. Belonging to school score showed that it is important for girls to feel “comfortable” early in their enrollment, while boys have reported a sense of alignment with SOC near graduation (Yamazaki et al., 2008). Although these experiences differed between boys and girls both genders showed similar trends. The short time students spent at school upon enrollment may have changed their sense of comfort and attachment, as they became more involved with their surroundings as their time at school gradually increased.

### 4.3. Differences in the Impact of the Pandemic on G1 and G2

Multiple regression analysis showed that the 2022 SOC score for G2 had a significantly stronger association with the baseline SOC, whereas G1 showed no significant association. According to Antonovsky, SOC is “a person’s orientation to the world of life”, or, in other words, a person’s view and attitude toward the world of life. The fact that pre-pandemic SOC tendencies did not carry over among G1 students suggests that their (G1) perception of school life and the living world may have changed before and after the pandemic. In other words, for G1, the pandemic had a major impact on their previous school life and its meaning, which was changed by the pandemic. As for other significant associations, a negative association was found between “stress related to schoolwork” and SOC, which can be due to the pressure of the impending college entrance exam. The largest coefficient β was for “belonging to school” (β = 0.517, *p* = 0.009). Experiencing a day when school was suddenly unavailable due to the closure may have increased the importance of school as a place of comfort outside the home, especially for G1 students, suggesting that school may function as a safe place.

For G2, the largest significant association with the dependent variable (2022 SOC total score) was the baseline SOC score. We inferred that this means that school life under the pandemic is the standard and default for them, indicating that, unlike G1, their “way of perceiving the world” has not changed significantly. Being a girl and experiencing family-related stress were negatively and significantly associated with the dependent variable. There are various indications of gender differences in SOC (Rivera et al., 2013), with some studies reporting higher SOC in boys (Nilsson et al., 2010). Family relationships also affect SOC (García-Moya et al., 2012; Omiya et al., 2021). It is possible that the reduced time spent at school due to fewer club activities and school events and the few experiences at home may have had a sensitive effect, particularly when students faced advancement to the third year of junior high school. Therefore, it is necessary to confirm the long-term effects of these factors.

### 4.4. Limitations

The study included only two grades in one integrated junior and senior high school in Tokyo, Japan, and only a few of the analyzed students could participate for all three or four years. Although it is a public school, it requires an “aptitude test” at the time of admission, and students are selected for admission. It cannot be ruled out that the results of the analysis may be characteristic of the school or grade level; therefore, they are difficult to generalize. However, we believe that it is valuable to have SOC longitudinal data on adolescents on a yearly basis and to be able to track changes in the generation that experienced COVID-19. To obtain universal findings, it is necessary to analyze data from several different types of schools and grades in the future. Additionally, despite being from the same school, there was a significant difference in the number of participants between G1 and G2, with G2 having more than twice the number of participants. G1 did not have many dropouts due to the four-year follow-up period. The cooperating schools had a capacity of 160 students per grade level, but the reason why 144 students agreed to participate in G2. This may be because the survey was started during the pandemic, and parents were more interested in their children’s mental health. In cases where parental consent was not obtained for either G1 or G2, the reasons for this were unknown. There may be serious problems in cases of nonparticipation or dropout from the beginning, which need to be examined in the future.

## 5. Conclusions

The baselines of SOC were tracked in 2019, 2020, and for either four or three years, respectively, for two groups of middle school students who were enrolled before COVID-19 (G1) and during the COVID-19 pandemic (G2).

At baseline, G2 had significantly higher SOC scores, but there was no significant difference between the two groups by 2022. In G1, there was a significant increase in comprehensibility and manageability from 2019 onwards, especially among girls. However, meaningfulness decreased from baseline in G2 for both boys and girls, possibly due to the pandemic. The scores on the school membership scale did not differ significantly by year in G1, but in G2 there was a significant decrease from baseline for both boys and girls regarding the parameter belonging to school. G2 may have found school life restricted by the pandemic to be different from what they expected, or may have had difficulty making sense of the various environmental and rule changes.

Multiple regression analysis with the 2022 SOC score as the dependent variable showed that G1 was not associated with baseline SOC, suggesting that the pandemic may have caused a change in their “way of perceiving the living world”. In G2, the baseline 2020 score and the dependent variable, the 2022 score, were significantly related, and 2022 could be regarded as an extension of 2020. From the above, it is believed that the pandemic has had various long-term effects on adolescents, and they need to be carefully observed considering their respective grades and gender. Meticulous care and support should be given to them and their families.

The results of the analysis of G2 suggest that the group who entered school after the pandemic may have a lower sense of meaningfulness. Antonovsky states that “participation in the formation of outcomes” is important for increasing the sense of meaningfulness. It is important for schools to consciously create opportunities for students to make their own decisions about school events and club activities.

The age group of G1 will be the generation graduating from school, and the analysis results suggest that their outlook the world and their lives may have changed. It would be desirable to conduct research over a 10-year period to explore the presence or absence of long-term effects on a generation that has experienced a complete change in their school life.

As mentioned above, adolescent SOC is said to be closely related to adult SOC. However, times are changing rapidly. The pandemic has caused the dissemination of information technology, completely altering the information environment surrounding children. Therefore, we believe that collection and analysis of data on the generation directly affected by the pandemic was significant.

## Figures and Tables

**Table 1:** Attributes and characteristics in 2 groups.

	G1: Students Admitted in 2018 (Before COVID-19)	G2: Students Admitted in 2020 (During COVID-19)	*t*-Test *p*-Value
Variables	n = 96	n = 144	
	N (%), Average Score, ±SD		
Gender Boy	49 (51.0)	73 (50.7)	0.273
Girl	47 (49.0)	71 (49.3)	(χ^2^-test)
Baseline (G1 = Year 2019, G2 = Year 2020)			
Subjective physical health (range 8–32)	24.10 ± 5.0	26.08 ± 4.1	0.001
Athens insomnia score (the higher the score, the more insomnia)	3.27 ± 2.5	2.99 ± 2.5	0.398
Sense of coherence (range 13–65) total score	39.40 ± 6.3	44.20 ± 7.7	<0.001
Comprehensibility	14.44 ± 3.2	16.00 ± 3.5	<0.001
Manageability	11.82 ± 2.7	13.33 ± 3.0	<0.001
Meaningfulness	13.16 ± 2.7	14.89 ± 2.8	<0.001
School membership scale			
Accepted by students	16.54 ± 2.7	17.16 ± 2.6	0.075
Accepted by teachers	18.47 ± 3.5	19.29 ± 3.5	0.074
Belonging to school	16.63 ± 3.0	17.38 ± 2.5	0.038
Year 2022			
Sense of coherence (range 13–65) total score	41.10 ± 7.4	41.72 ± 6.9	0.992
Comprehensibility	15.14 ± 3.2	15.40 ± 3.4	0.652
Manageability	13.00 ± 2.7	13.02 ± 2.9	0.957
Meaningfulness	13.00 ± 2.8	13.21 ± 2.8	0.570
School membership scale			
Accepted by students	16.13 ± 3.0	16.86 ± 2.9	0.065
Accepted by teachers	19.07 ± 4.1	19.07 ± 4.4	1.000
Belonging to school	15.75 ± 3.4	16.22 ± 2.8	0.247
Stress experience scale			
Stress for teachers	6.17 ± 2.1	6.04 ± 1.8	0.640
Stress from club activities	6.86 ± 2.6	7.02 ± 2.6	0.686
Stress related to schoolwork	8.97 ± 3.3	9.06 ± 3.4	0.858
Stress related to friends	5.36 ± 1.1	5.11 ± 0.4	0.211
Family-related stress	5.9 ± 3.0	6.52 ± 3.4	0.207

**Table 2:** Comparison of gender groups on the SOC total score and three subscales: one-way ANOVA with repeated measures over years (paired factors).

	G1: Students Admitted in 2018 (Before COVID-19) n = 96								G2: Students Admitted in 2020 (During COVID-19) n = 114								
	Boy n = 49			Girl n = 47					Boy n = 73				Girl n = 71				
Variables	Average Score ±SD	Year Bonferroni Test *p*-Value		Average Score ± SD	Year Bonferroni Test *p*-Value			Male/Female *t*-Test *p*-Value	Average Score ± SD	Year Bonferroni Test *p*-Value			Average Score ± SD	Year Bonferroni Test *p*-Value			Male/ Female *t*-Test *p*-Value
Sense of coherence (SOC) total score (range 13–65)																	
Year 2019	39.59 ± 6.0			39.25 ± 6.8	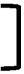	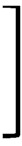	<0.001	0.802	-				-				-
2020	40.57 ± 7.2			43.80 ± 8.1				0.043	46.05 ± 6.9				42.40 ± 8.0	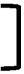	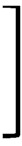	0.008	0.010
2021	40.77 ± 7.4			42.72 ± 8.0			0.023	0.260	44.30 ± 7.8				40.20 ± 8.1				0.002
2022	41.31 ± 8.1			42.05 ± 6.9				0.409	44.05 ± 7.5				39.24 ± 7.0			<0.001	<0.001
SOC subcomponents																	
Comprehensibility																	
Year 2019	14.92 ± 3.3			13.95 ± 3.0	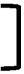	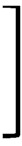	0.001	0.075	-				-				-
2020	15.02 ± 3.7			16.07 ± 3.5				0.599	16.95 ± 3.5				15.03 ± 3.4	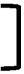	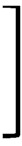	0.043	0.005
2021	15.16 ± 3.4			15.75 ± 3.4			0.013	0.484	17.12 ± 3.7				14.24 ± 3.5				<0.001
2022	14.93 ± 3.6			± 3.0				0.547	16.70 ± 3.4				14.13 ± 3.0			0.029	<0.001
Manageability																	
Year 2019	11.77 ± 2.6	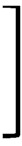	0.027	11.87 ± 2.9	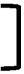	0.014		0.858	-				-				-
2020	12.96 ± 2.4			13.24 ± 2.9				0.345	14.07 ± 3.0				12.58 ± 2.8				0.002
2021	13.02 ± 2.7			13.08 ± 2.9				0.928	13.94 ± 3.3				12.73 ± 3.0				0.019
2022	12.93 ± 3.0			13.08 ± 2.5				0.297	13.83 ± 2.8				12.19 ± 2.8				<0.001
Meaningfulness																	
Year 2019	12.90 ± 2.8			13.43 ± 2.6				0.343	-				-				-
2020	12.59 ± 2.5			14.50 ± 2.9				0.001	15.03 ± 2.6	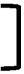	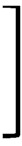	<0.001	14.75 ± 3.1	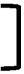	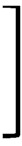	<0.001	0.675
2021	12.59 ± 3.4			13.89 ± 3.1				0.061	13.26 ± 3.3				13.19 ± 3.0				0.894
2022	12.42 ± 2.5			13.60 ± 2.8				0.089	13.52 ± 2.8			0.001	12.90 ± 2.8			0.001	<0.001

Blank spaces indicate not significant.

**Table 3:** Comparison of the three subscales of school membership by gender: one-way ANOVA with repeated measures over years (paired factors).

	G1: Students Admitted in 2018 (Before COVID-19) n = 96					G2: Students Admitted in 2020 (During COVID-19) n = 144								
	Boy n = 49		Girl n = 47			Boy n = 73				Girl n = 71				
Variables	Average Score ± SD	Year Bonferroni Test *p*-Value	Average Score ± SD	Year Bonferroni Test *p*-Value	Male/ Female *T*-Test *p*-Value	Average Score ± SD	Year Bonferroni Test *p*-Value			Average Score ± SD	Year Bonferroni Test *p*-Value			Male/ Female *T*-Test *p*-Value
School membership scale														
Accepted by students														
Year 2019	15.76 ± 2.6		17.35 ± 2.5		0.004	-				-				-
2020	15.41 ± 3.1		16.98 ± 2.7		0.013	16.77 ± 2.7				17.56 ± 2.4				0.067
2021	15.64 ± 2.9		16.75 ± 2.7		0.066	16.33 ± 3.3				17.04 ± 3.1				0.180
2022	15.41 ± 3.4		16.88 ± 2.6		0.060	16.72 ± 3.1				17.00 ± 2.7				0.574
Accepted by teachers														
Year 2019	17.80 ± 3.4		19.17 ± 3.3		0.052	-				-				-
2020	18.08 ± 4.3		20.12 ± 3.5		0.016	19.18 ± 3.4				19.40 ± 3.5				0.702
2021	18.02 ± 4.5		19.62 ± 3.8		0.082	18.75 ± 4.1				18.61 ± 4.5				0.840
2022	17.89 ± 4.5		20.29 ± 3.5		0.023	19.16 ± 4.4				18.97 ± 4.4				0.801
Belonging to school														
Year 2019	16.20 ± 3.1		17.09 ± 2.9		0.153	-				-				-
2020	15.73 ± 3.1		16.64 ± 3.1		0.162	17.45 ± 2.5	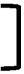	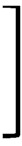	0.010	17.30 ± 2.5	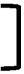	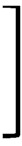	0.001	0.710
2021	15.77 ± 2.7		16.91 ± 2.9		0.059	16.53 ± 2.9				16.35 ± 3.2				0.637
2022	14.89 ± 3.4		16.65 ± 3.3		0.047	16.43 ± 2.8			0.002	15.99 ± 2.8			<0.001	0.308

Blank spaces indicate not significant.

**Table 4:** Multiple regression analysis with 2022 SOC total score as the dependent variable (2 groups).

Variables	G1: Students Admitted in 2018 (Before COVID-19) n = 96						G2: Students Admitted in 2020 (During COVID-19) n = 144					
	Correlation Analysis		Multivariate				Correlation Analysis		Multivariate			
	Spearman ρ	*p*-Value	Coefficient β	*p*-Value	Standard Error	95%CI	Spearman ρ	*p*-Value	Coefficient β	*p*-Value	Standard Error	95%CI
Gender (Boy = 0, Girl = 1)	0.173	0.096	0.138	0.286	1.950	−1.822–6.032	−0.391	<0.001	−0.212	0.009	1.255	−5.849–−0.844
[Baseline]												
(G1 = year 2018, G2 = year 2020)												
SOC total score (range: 13–65)	0.089	0.252	−0.065	0.664	0.157	−0.385–0.248	0.672	<0.001	0.369	0.001	0.109	0.154–0.593
Subjective physical health	0.207	0.059	−0.065	0.066	0.212	−0.027–0.826	0.361	<0.001	0.071	0.490	0.199	−0.260–0.538
Athens insomnia score	−0.077	0.282	0.279	0.510	0.419	−0.566–1.123	0.491	<0.001	0.009	0.928	0.305	−0.580–0.635
[Measurement score in 2020]			0.104									
School membership scale												
Accepted by students	0.260	0.024		0.056	0.528	−2.100–0.027	0.360	<0.001	−0.104	0.413	0.326	−0.917–0.381
Accepted by teachers	0.302	0.010	−0.418	0.468	0.354	−0.454–0.972	0.491	<0.001	0.065	0.639	0.239	−0.364–0.589
Belonging to school	0.465	0.000	0.14	0.009	0.411	0.293–1.950	0.481	0.001	0.204	0.096	0.326	−0.100–1.199
Stress experience scale			0.516									
Stress for teachers	−0.231	0.041		0.849	0.536	−0.978–1.183	−0.213	0.025	0.041	0.619	0.461	−0.689–1.150
Stress from club activities	−0.228	0.043	0.029	0.287	0.426	−1.318–0.399	−0.350	0.001	0.043	0.637	0.332	−0.505–0.819
Stress related to schoolwork	−0.461	0.000	−0.152	0.010	0.337	−1.582–−0.226	−0.528	<0.001	−0.128	0.213	0.246	−0.803–0.182
Stress related to friends	−0.360	0.003	−0.393	0.669	1.102	−2.694–1.745	−0.118	0.142	−0.077	0.350	1.704	−4.999–1.793
Family-related stress	−0.210	0.057	−0.072	0.672	0.359	−0.571–0.877	−0.441	<0.001	−0.230	0.015	0.231	−1.033–−0.114
Adjusted R^2^	0.323						0.543					
	*p* = 0.002						*p* < 0.001					

## Data Availability

In some cases, the corresponding author may make the data from this study available upon request. However, because the survey included highly sensitive information regarding parents and children enrolled in Tokyo high schools, as well as details regarding autism in relation to legal minors, the statistics are not made public. Even anonymous disclosure of raw data is prohibited for the schools involved.

## Abbreviations

The following abbreviations are used in this manuscript:

SOC	Sense of coherence
