# Correction: Omiya and Deguchi (2025). Adolescent Sense of Coherence over a Four-Year Period and the Pandemic: Junior and Senior High School Students Enrolled Before and After the Pandemic Broke out in Japan. *Behavioral Sciences*, *15*(4), 504

**DOI:** 10.3390/bs15091174

**Published:** 2025-08-29

**Authors:** Tomoko Omiya, Naoko Kumada Deguchi

**Affiliations:** 1 Public Health Nursing, Institute of Medicine, University of Tsukuba, Tsukuba 305-8575, Japan; 2 Faculty of Education, Shizuoka University, Shizuoka 422-8017, Japan

There was an error in the original publication (Omiya & Deguchi, 2025).

The number of samples in the abstract should be “96” instead of “97”.

A correction has been made to the Abstract:

Based on Antonovsky’s theory, we explored the importance of adolescent sense of coherence (SOC) in coping with stress and how it was affected by the pandemic. Using longitudinal data from junior and senior high school students in urban areas in Japan, we examined the trends in SOC and factors related to SOC in students enrolled before and after the COVID-19 pandemic. With the cooperation of the Tokyo Metropolitan Secondary Education School, we surveyed 96 students who enrolled in 2018 (G1) and 144 students who enrolled in 2020 (G2). Four surveys were conducted for G1 and three for G2. Survey items included SOC, psychosomatic symptoms scale, Athens insomnia scale, school belonging scale, and stress experience scale. We followed the trends in SOC scores by gender and performed *t*-tests and multiple regression analysis. G2 had higher baseline SOC scores than G1, but the significant difference between the two groups disappeared by 2022. From 2019, comprehensibility and manageability significantly increased in G1 for girls, but meaningfulness decreased in G2 for both boys and girls. Multiple regression analysis showed no correlation between baseline SOC and SOC in 2022 in G1, which differed from G2, suggesting that the pandemic may have changed their perception of the world.

There is a typo in the Section 1, in which “accoount” should be “account”.

A correction has been made to the Section 1 in Paragraph 5:

DeVylder et al. (2024) reported that mental disorders in adolescents, which had decreased before the pandemic, began to rise again at the same time as the pandemic, despite the low infection rate of COVID-19 in Tokyo, Japan. They attributed this to COVID-19-related social and environmental factors. In addition, Raney et al. (2022) revealed that children with more adverse experiences related to the pandemic had longer screen time and less physical activity. Orben et al. (2020) stated that although the measures to maintain physical distance from others due to the pandemic are temporary, the impact of maintaining physical distance for several months may be stronger on young people’s development than on adults, as this account constitutes a large proportion of their lives during a sensitive period of development. They were concerned about the impact of the pandemic on young people’s development. In addition, many studies on adolescents were conducted during the first months of the pandemic (i.e., through the summer of 2020) (Barendse et al., 2023; Hafstad et al., 2021). However, the impact of the pandemic on adolescents may evolve over time, influenced by factors such as the reopening of schools or changes in social distance regulations (Hosozawa et al., 2024), and should be tracked over time. Furthermore, the significance of the pandemic and school closure is likely to be different for the group that experienced school closure while still in school and the group entering school in April 2020, where the pandemic coincided with their enrollment. Those still in school were unable to attend important ceremonies and had difficulty building relationships with friends through online classes and other means. If the impact of the pandemic differs between the group that experienced the pandemic after enrollment and the group that enrolled after the pandemic, interventions and support will only be effective if they reflect the circumstances of each group. However, no studies have been conducted from such a perspective. It is important to clarify the characteristics of these two groups to obtain suggestions for support for each.

There are mistakes in the grades of students in Section 2.1. They should be “from eighth through eleventh grade” and “from seventh to ninth grade”.

A correction has been made to Section 2.1, Paragraph 2:

The baseline for G1 was spring 2019, while for G2 it was summer 2020. G1 data were collected through four surveys over four years, from eighth through eleventh grade, whereas G2 data were gathered through three surveys over three years, from seventh to ninth grade.

The range of Cronbach’s alpha coefficients for the SOC-13 scale in Section 2.3 should be “0.84 to 0.87”, and in Section 2.4, it should be “0.81 to 0.89”.

A correction has been made to Section 2.3, Paragraph 1:

We used the sense of coherence 13-item scale (SOC-13), a shortened Japanese version of the original SOC 29-item scale developed by Antonovsky. The reliability and validity of the SOC-13 were confirmed by Togari and Yamazaki through a survey conducted in Japan (Togari et al., 2007). The SOC-13 comprises three subscales: comprehension (five items), manageability (four items), and meaningfulness (four items). In the SOC-13 scale, items are rated on a 5-point Likert scale. Higher scores indicate a greater ability to maintain health and cope with stress. G1 was measured four times from 2019 to 2022, and G2 was measured three times from 2020 to 2022. The Cronbach’s SOC-13 alphas in this study ranged from 0.84 to 0.87.

A correction has been made to Section 2.4, Paragraph 1:

We used the school membership scale, a shortened Japanese version of the psychological sense of school membership scale (Goodenow, 1993) developed by Goodenow, an educational psychologist (Togari et al., 2011). The scale consists of three components—student acceptance (five items), teacher acceptance (four items), and sense of belonging (four items)—with a total of 13 items measured using a five-item method. The reliability and validity of the Japanese version of the scale have been confirmed (Soldatos et al., 2000). Higher total scores indicated a greater commitment to school life. G1 was measured four times from 2019 to 2022, and G2 was measured three times from 2020 to 2022. The Cronbach’s alpha in this study ranged from 0.81 to 0.89.

The number of items in Section 2.5 should be “two” instead of “eight”, and the first category should be “diarrhea/constipation” instead of “diarrhea, constipation”.

A correction has been made to Section 2.5, Paragraph 1:

They were asked about six psychosomatic symptoms as perceived by Ben-Sira (1982) (headache, abdominal pain, difficulty sleeping, palpitations, vertigo or dizziness, irritability) and two items relating to diarrhea/constipation and body pain (e.g., shoulder pain). Respondents were asked to answer “always”, “sometimes”, “not often”, and “not at all”, using a 4-point scale, with 1 to 4 points given for each. Scores range from 8 to 32 points, with higher scores indicating better physical condition.

A sentence stating, “To address missing data without reducing the sample size, multiple imputation (MI) was conducted.” should be added to Section 2.6.

A correction has been made to Section 2.6, Paragraph 4:

Multiple regression analysis was performed for each group with the 2022 SOC total score as the dependent variable. To address missing data without reducing the sample size, multiple imputation (MI) was conducted. The explanatory variables were the baseline SOC total score, psychosomatic illness, Athens insomnia scale score, and the 2022 sense of school belonging scale and stress experience scale, after confirming that the Spearman’s ρ correlation with the dependent variable was significant in one or both groups. SPSS version 29 was used for statistical analysis.

The word “similar” in Section 3.1 should be “different”. There is also a mistake in Paragraph 4, in which “20.6” should be “2.6”.

A correction has been made to Section 3.1, Paragraph 2:

As shown in Table 2, A one-way ANOVA (with Bonferroni’s for subsequent tests) with repeated measures (factors with correspondence) by year, group, and gender was performed for the SOC total score and the three subscales. The results showed that, in G1, the SOC total score for girls was different in 2019 and 2020 (39.2 ± 6.8 and 43.8 ± 8.1, *p* < 0.001), and in 2019 and 2021 (42.7 ± 8.0, *p* = 0.023), showing significant differences. For the subscales, significant differences were found for comprehensibility in 2019 and 2020 (14.0 ± 3.0 and 16.1 ± 3.5, *p* = 0.001) and 2019 and 2021 (15.8 ± 3.4, *p* = 0.013) for girls. For manageability, girls showed a significant difference in 2019 (11.9 ± 2.9) and 2020 (13.2 ± 2.9) (*p* = 0.014). Boys showed a significant difference in 2019 (11.7 ± 2.6) and 2021 (13.0 ± 2.7) (*p* = 0.027).

A correction has been made to Section 3.1, Paragraph 4:

Looking at year-to-year trends by gender for G2, girls had the highest baseline scores in 2020 for the SOC total score, as well as the comprehensibility and meaningfulness subscales, with significant differences observed between 2020 and 2021, and between 2020 and 2022 (*p* < 0.001–0.043). Significant differences were found between 2020 and 2021, and between 2020 and 2022 (*p* < 0.001 to 0.043). For boys, significant differences were found only in the meaningfulness subscale, with higher scores in 2020 (15.0 ± 2.6) compared to 2021 (13.3 ± 3.3, *p* < 0.001) and 2022 (13.5 ± 2.8, *p* = 0.001). Regarding the comparison of G2 scores, no gender differences were found in the meaningfulness subscale in 2020 and 2021. However, all other measured variables were significantly higher in boys (*p* < 0.001 to 0.019).

In Section 3.2, the β of G1 should be “0.516” instead of “0.517”.

A correction has been made to Section 3.2, Paragraph 1:

Table 4 presents the results of the multiple regression analysis using the 2022 SOC total score as the dependent variable. In G1, the sense of belonging (β = 0.516, *p* = 0.009), a subscale of the sense of school belonging scale, was positively and significantly associated with the dependent variable, while academic stress was negatively and significantly associated (β = −0.393, *p* = 0.010). In G2, the baseline total SOC score showed a significant positive association with the dependent variable (β = 0.370, *p* = 0.001), while it negatively and significantly correlated with the covariates gender, with girls having lower SOC (β = −0.212, *p* = 0.009) and family stress (β = −0.230, *p* = 0.015).

There are two grammatical mistakes in Section 4.4. Add “it” after “although” and add “why” after “reason”.

A correction has been made to Section 4.4, Paragraph 1:

The study included only two grades in one integrated junior and senior high school in Tokyo, Japan, and only a few of the analyzed students could participate for all three or four years. Although it is a public school, it requires an “aptitude test” at the time of admission, and students are selected for admission. It cannot be ruled out that the results of the analysis may be characteristic of the school or grade level; therefore, they are difficult to generalize. However, we believe that it is valuable to have SOC longitudinal data on adolescents on a yearly basis and to be able to track changes in the generation that experienced COVID-19. To obtain universal findings, it is necessary to analyze data from several different types of schools and grades in the future. Additionally, despite being from the same school, there was a significant difference in the number of participants between G1 and G2, with G2 having more than twice the number of participants. G1 did not have many dropouts due to the four-year follow-up period. The cooperating schools had a capacity of 160 students per grade level, but the reason why 144 students agreed to participate in G2. This may be because the survey was started during the pandemic, and parents were more interested in their children’s mental health. In cases where parental consent was not obtained for either G1 or G2, the reasons for this were unknown. There may be serious problems in cases of nonparticipation or dropout from the beginning, which need to be examined in the future.

There is a typo in the Section 5, in which “he” should be “they”.

A correction has been made to the Section 5, Paragraph 2:

At baseline, G2 had significantly higher SOC scores, but there was no significant difference between the two groups by 2022. In G1, there was a significant increase in comprehensibility and manageability from 2019 onwards, especially among girls. However, meaningfulness decreased from baseline in G2 for both boys and girls, possibly due to the pandemic. The scores on the school membership scale did not differ significantly by year in G1, but in G2 there was a significant decrease from baseline for both boys and girls regarding the parameter belonging to school. G2 may have found school life restricted by the pandemic to be different from what they expected, or may have had difficulty making sense of the various environmental and rule changes.

The third approval year in the IRB statement should be 2021.

A correction has been made to the Institutional Review Board Statement:

This study was approved by the Medical Ethics Review Committee of the University of Tsukuba (approval numbers: 1343, 1343-1 and 2), approval dates: 30 January 2019; 10 June 2020, and 8 June 2021.

In the original publication, there was a mistake in Tables 1–4 as published. In Table 1, a typographical error in the row labels was corrected, changing “Boye” to “Boy.” In Table 2, the sample size (n) for Group 1 (G1) was added, and the sample size for Group 2 (G2) was corrected to 144. Table 3 was updated to replace the gender category labels “male” and “female” with “boy” and “girl,” and the sample sizes were corrected to n = 96 for G1 and n = 144 for G2. Similarly, in Table 4, gender labels were changed for consistency, and several statistical values were revised to reflect accurate calculations.

The corrected Table 1, Table 2, Table 3 and Table 4 appear below.

**Table 1:** Attributes and characteristics in 2 groups.

	G1: Students Admitted in 2018 (Before COVID-19)	G2: Students Admitted in 2020 (During COVID-19)	*t*-Test *p*-Value
Variables	n = 96	n = 144	
	N (%), Average Score, ±SD		
Gender Boy	49 (51.0)	73 (50.7)	0.273
Girl	47 (49.0)	71 (49.3)	(χ^2^-test)
Baseline (G1 = Year 2019, G2 = Year 2020)			
Subjective physical health (range 8–32)	24.10 ± 5.0	26.08 ± 4.1	0.001
Athens insomnia score (the higher the score, the more insomnia)	3.27 ± 2.5	2.99 ± 2.5	0.398
Sense of coherence (range 13–65) total score	39.40 ± 6.3	44.20 ± 7.7	<0.001
Comprehensibility	14.44 ± 3.2	16.00 ± 3.5	<0.001
Manageability	11.82 ± 2.7	13.33 ± 3.0	<0.001
Meaningfulness	13.16 ± 2.7	14.89 ± 2.8	<0.001
School membership scale			
Accepted by students	16.54 ± 2.7	17.16 ± 2.6	0.075
Accepted by teachers	18.47 ± 3.5	19.29 ± 3.5	0.074
Belonging to school	16.63 ± 3.0	17.38 ± 2.5	0.038
Year 2022			
Sense of coherence (range 13–65) total score	41.10 ± 7.4	41.72 ± 6.9	0.992
Comprehensibility	15.14 ± 3.2	15.40 ± 3.4	0.652
Manageability	13.00 ± 2.7	13.02 ± 2.9	0.957
Meaningfulness	13.00 ± 2.8	13.21 ± 2.8	0.570
School membership scale			
Accepted by students	16.13 ± 3.0	16.86 ± 2.9	0.065
Accepted by teachers	19.07 ± 4.1	19.07 ± 4.4	1.000
Belonging to school	15.75 ± 3.4	16.22 ± 2.8	0.247
Stress experience scale			
Stress for teachers	6.17 ± 2.1	6.04 ± 1.8	0.640
Stress from club activities	6.86 ± 2.6	7.02 ± 2.6	0.686
Stress related to schoolwork	8.97 ± 3.3	9.06 ± 3.4	0.858
Stress related to friends	5.36 ± 1.1	5.11 ± 0.4	0.211
Family-related stress	5.9 ± 3.0	6.52 ± 3.4	0.207

**Table 2:** Comparison of gender groups on the SOC total score and three subscales: one-way ANOVA with repeated measures over years (paired factors).

	G1: Students Admitted in 2018 (Before COVID-19) n = 96								G2: Students Admitted in 2020 (During COVID-19) n = 114								
	Boy n = 49			Girl n = 47					Boy n = 73				Girl n = 71				
Variables	Average Score ±SD	Year Bonferroni Test *p*-Value		Average Score ± SD	Year Bonferroni Test *p*-Value			Male/Female *t*-Test *p*-Value	Average Score ± SD	Year Bonferroni Test *p*-Value			Average Score ± SD	Year Bonferroni Test *p*-Value			Male/ Female *t*-Test *p*-Value
Sense of coherence (SOC) total score (range 13–65)																	
Year 2019	39.59 ± 6.0			39.25 ± 6.8	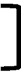	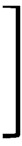	<0.001	0.802	-				-				-
2020	40.57 ± 7.2			43.80 ± 8.1				0.043	46.05 ± 6.9				42.40 ± 8.0	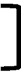	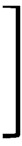	0.008	0.010
2021	40.77 ± 7.4			42.72 ± 8.0			0.023	0.260	44.30 ± 7.8				40.20 ± 8.1				0.002
2022	41.31 ± 8.1			42.05 ± 6.9				0.409	44.05 ± 7.5				39.24 ± 7.0			<0.001	<0.001
SOC subcomponents																	
Comprehensibility																	
Year 2019	14.92 ± 3.3			13.95 ± 3.0	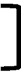	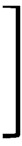	0.001	0.075	-				-				-
2020	15.02 ± 3.7			16.07 ± 3.5				0.599	16.95 ± 3.5				15.03 ± 3.4	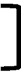	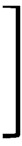	0.043	0.005
2021	15.16 ± 3.4			15.75 ± 3.4			0.013	0.484	17.12 ± 3.7				14.24 ± 3.5				<0.001
2022	14.93 ± 3.6			± 3.0				0.547	16.70 ± 3.4				14.13 ± 3.0			0.029	<0.001
Manageability																	
Year 2019	11.77 ± 2.6	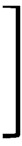	0.027	11.87 ± 2.9	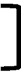	0.014		0.858	-				-				-
2020	12.96 ± 2.4			13.24 ± 2.9				0.345	14.07 ± 3.0				12.58 ± 2.8				0.002
2021	13.02 ± 2.7			13.08 ± 2.9				0.928	13.94 ± 3.3				12.73 ± 3.0				0.019
2022	12.93 ± 3.0			13.08 ± 2.5				0.297	13.83 ± 2.8				12.19 ± 2.8				<0.001
Meaningfulness																	
Year 2019	12.90 ± 2.8			13.43 ± 2.6				0.343	-				-				-
2020	12.59 ± 2.5			14.50 ± 2.9				0.001	15.03 ± 2.6	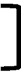	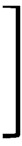	<0.001	14.75 ± 3.1	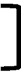	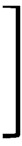	<0.001	0.675
2021	12.59 ± 3.4			13.89 ± 3.1				0.061	13.26 ± 3.3				13.19 ± 3.0				0.894
2022	12.42 ± 2.5			13.60 ± 2.8				0.089	13.52 ± 2.8			0.001	12.90 ± 2.8			0.001	<0.001

Blank spaces indicate not significant.

**Table 3:** Comparison of the three subscales of school membership by gender: one-way ANOVA with repeated measures over years (paired factors).

	G1: Students Admitted in 2018 (Before COVID-19) n = 96					G2: Students Admitted in 2020 (During COVID-19) n = 144								
	Boy n = 49		Girl n = 47			Boy n = 73				Girl n = 71				
Variables	Average Score ± SD	Year Bonferroni Test *p*-Value	Average Score ± SD	Year Bonferroni Test *p*-Value	Male/ Female *T*-Test *p*-Value	Average Score ± SD	Year Bonferroni Test *p*-Value			Average Score ± SD	Year Bonferroni Test *p*-Value			Male/ Female *T*-Test *p*-Value
School membership scale														
Accepted by students														
Year 2019	15.76 ± 2.6		17.35 ± 2.5		0.004	-				-				-
2020	15.41 ± 3.1		16.98 ± 2.7		0.013	16.77 ± 2.7				17.56 ± 2.4				0.067
2021	15.64 ± 2.9		16.75 ± 2.7		0.066	16.33 ± 3.3				17.04 ± 3.1				0.180
2022	15.41 ± 3.4		16.88 ± 2.6		0.060	16.72 ± 3.1				17.00 ± 2.7				0.574
Accepted by teachers														
Year 2019	17.80 ± 3.4		19.17 ± 3.3		0.052	-				-				-
2020	18.08 ± 4.3		20.12 ± 3.5		0.016	19.18 ± 3.4				19.40 ± 3.5				0.702
2021	18.02 ± 4.5		19.62 ± 3.8		0.082	18.75 ± 4.1				18.61 ± 4.5				0.840
2022	17.89 ± 4.5		20.29 ± 3.5		0.023	19.16 ± 4.4				18.97 ± 4.4				0.801
Belonging to school														
Year 2019	16.20 ± 3.1		17.09 ± 2.9		0.153	-				-				-
2020	15.73 ± 3.1		16.64 ± 3.1		0.162	17.45 ± 2.5	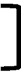	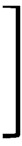	0.010	17.30 ± 2.5	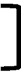	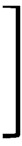	0.001	0.710
2021	15.77 ± 2.7		16.91 ± 2.9		0.059	16.53 ± 2.9				16.35 ± 3.2				0.637
2022	14.89 ± 3.4		16.65 ± 3.3		0.047	16.43 ± 2.8			0.002	15.99 ± 2.8			<0.001	0.308

Blank spaces indicate not significant.

**Table 4:** Multiple regression analysis with 2022 SOC total score as the dependent variable (2 groups).

Variables	G1: Students Admitted in 2018 (Before COVID-19) n = 96						G2: Students Admitted in 2020 (During COVID-19) n = 144					
	Correlation Analysis		Multivariate				Correlation Analysis		Multivariate			
	Spearman ρ	*p*-Value	Coefficient β	*p*-Value	Standard Error	95%CI	Spearman ρ	*p*-Value	Coefficient β	*p*-Value	Standard Error	95%CI
Gender (Boy = 0, Girl = 1)	0.173	0.096	0.138	0.286	1.950	−1.822–6.032	−0.391	<0.001	−0.212	0.009	1.255	−5.849–−0.844
[Baseline]												
(G1 = year 2018, G2 = year 2020)												
SOC total score (range: 13–65)	0.089	0.252	−0.065	0.664	0.157	−0.385–0.248	0.672	<0.001	0.369	0.001	0.109	0.154–0.593
Subjective physical health	0.207	0.059	−0.065	0.066	0.212	−0.027–0.826	0.361	<0.001	0.071	0.490	0.199	−0.260–0.538
Athens insomnia score	−0.077	0.282	0.279	0.510	0.419	−0.566–1.123	0.491	<0.001	0.009	0.928	0.305	−0.580–0.635
[Measurement score in 2020]			0.104									
School membership scale												
Accepted by students	0.260	0.024		0.056	0.528	−2.100–0.027	0.360	<0.001	−0.104	0.413	0.326	−0.917–0.381
Accepted by teachers	0.302	0.010	−0.418	0.468	0.354	−0.454–0.972	0.491	<0.001	0.065	0.639	0.239	−0.364–0.589
Belonging to school	0.465	0.000	0.14	0.009	0.411	0.293–1.950	0.481	0.001	0.204	0.096	0.326	−0.100–1.199
Stress experience scale			0.516									
Stress for teachers	−0.231	0.041		0.849	0.536	−0.978–1.183	−0.213	0.025	0.041	0.619	0.461	−0.689–1.150
Stress from club activities	−0.228	0.043	0.029	0.287	0.426	−1.318–0.399	−0.350	0.001	0.043	0.637	0.332	−0.505–0.819
Stress related to schoolwork	−0.461	0.000	−0.152	0.010	0.337	−1.582–−0.226	−0.528	<0.001	−0.128	0.213	0.246	−0.803–0.182
Stress related to friends	−0.360	0.003	−0.393	0.669	1.102	−2.694–1.745	−0.118	0.142	−0.077	0.350	1.704	−4.999–1.793
Family-related stress	−0.210	0.057	−0.072	0.672	0.359	−0.571–0.877	−0.441	<0.001	−0.230	0.015	0.231	−1.033–−0.114
Adjusted R^2^	0.323						0.543					
	*p* = 0.002						*p* < 0.001					

The authors state that the scientific conclusions are unaffected. This correction was approved by the Academic Editor. The original publication has also been updated.
